# Supplementary material for: DNA punch cards for storing data on native DNA sequences via enzymatic nicking
Source: Nat Commun. 2020 Apr 8;11:1742. doi: 10.1038/s41467-020-15588-z (PMC7142088; doi:10.1038/s41467-020-15588-z)
Supplement: Supplementary file 3 — Description of Additional Supplementary Files [file 41467_2020_15588_MOESM3_ESM.pdf]

Title: Supplementary Movie 1.
Description: The video shows the translocation of a 30 bp DNA fragment, nicked in the middle, through a MoS_2 nanopore. Further details and findings may be found in the Supplementary Information, under section B.7, Solid state nanopore simulations.
